# Advancing European paediatric research - the contribution of the Innovative Health Initiative

**DOI:** 10.3389/fmed.2025.1553448

**Published:** 2025-03-14

**Authors:** Nathalie Seigneuret

**Affiliations:** Innovative Health Initiative, Brussels, Belgium

**Keywords:** research and innovation, medicines development, collaboration, public private partnerships, medical product development

## Abstract

Children deserve health solutions, including medicines, medical devices and diagnostics, that are adapted to their needs. They should not be left behind when it comes to benefitting from innovations. The introduction of paediatric legislation in the EU and US in the 2000s dramatically changed the regulatory environment by fostering the development of medicines for children. However, the development of paediatric medicines remains challenging, and many needs remain unmet. When it comes to medical devices and *in vitro* diagnostics (IVDs), very few are designed and intended specifically for use in children, leading doctors to use adult devices and adapt them to fit children. To address the scientific, technical, and operational challenges related to paediatric development, multi-stakeholder collaboration is key. The European public-private partnerships the Innovative Health Initiative (IHI), and its predecessor the Innovative Medicines Initiative (IMI), contribute to advancing paediatric research by bringing together the private health industry sectors and public partners including academia, healthcare providers, patients and carers, regulators, and health technology assessment bodies. Several of their large collaborative research projects have already produced significant results that are optimising the development of paediatric medicines. This article looks at these achievements and discusses opportunities for further public-private collaborative research to boost the development of innovative health solutions that address specifically all children’s needs.

## 1 Introduction

Children deserve health solutions, including medicines, medical devices, and diagnostics, that are adapted to their needs. They should not be left behind when it comes to benefitting from innovations.

The introduction of paediatric legislation in the EU and the US in the 2000s dramatically changed the regulatory environment by fostering the development of medicines for children. For instance, the EU Paediatric Regulation has had a considerable impact, with 101 paediatric medicines and 99 new paediatric indications authorised centrally by 2016, over 1,000 paediatric investigation plans (PIPs) agreed in 2018, and an increase of almost 50% in the number of clinical trials that included child participants (1). However, developing paediatric medicines remains challenging, and many needs remain unmet. Few medical devices and *in vitro* diagnostics (IVDs) are designed and intended for use in paediatrics, leading doctors to use adult devices and adapt them to fit children.

To address the scientific, technical, and operational challenges related to paediatric development, multi-stakeholder collaboration is key. The European public–private partnerships, the Innovative Health Initiative (IHI), and its predecessor, the Innovative Medicines Initiative (IMI), contribute to advancing paediatric research by bringing together the private health industry sectors and public partners including academia, healthcare providers, patients and carers, regulators, and health technology assessment bodies. Several of their large collaborative precompetitive research projects have already produced significant results that are optimising the development of paediatric medicines.

## 2 Better paediatric clinical trials

### 2.1 Clinical networks

Due to the limited number of children affected, conducting appropriate paediatric clinical trials is challenging both scientifically and operationally. The IMI project conect4children^1^ was conceived to overcome the fragmented situation in Europe by setting up a pan-European paediatric clinical trial network capable of supporting all types of clinical trials used for regulatory purposes. Having such a network is critical to speeding up recruitment, running better-quality paediatric clinical trials, and coping with an increasing number of trials as a result of the growing number of PIPs. The project has built a network that acts as a single point of contact for all industry and non-industry sponsors and includes 20 national hubs across 21 European countries, providing access to over 250 clinical sites. Aligned processes have been implemented across the entire network to guarantee efficiency and quality. To ensure that all study sites and site personnel are well-trained, a training academy platform was created offering multiple short and advanced educational courses in various aspects of paediatric development. To promote the standardisation and harmonisation of paediatric clinical trial data, thereby ensuring interoperability, the paediatric user guide was developed (2). The project also contributed to a recipe to make trial data more findable, accessible, interoperable, and reusable (FAIR) by defining study and protocol-level (meta)data commonly collected in paediatric clinical trials (3). A process was designed for getting advice from a large pool of clinical and methodological experts, as well as input from children/parents on study designs and their feasibility. This is particularly important to help any sponsor to design high-quality, innovative, and feasible study protocols. Together, all these achievements create an efficient infrastructure capable of conducting better-designed paediatric clinical trials and thereby boosting the development of new medicines for children. The project established the not-for-profit foundation conect4children Stichting^2^ to carry forward the network and its services so that it can continue to contribute to Europe’s attractiveness for paediatric clinical trials in the future.

### 2.2 Innovative trial designs

Innovative designs including platform trials hold out promise as a way to optimise clinical trials, but whilst these have great potential, they are rarely used in paediatric clinical developments.

The IMI projects INNODIA^3^ and INNODIA HARVEST^4^ have built a clinical network on type 1 diabetes (T1D) to perform complex observational and clinical trials in T1D prevention or intervention. The network of accredited T1D clinical centres offers a standardised trial setting that is trained and ready to test innovative designs. The INNODIA Master Protocol was developed to explore the use of single or combinations of medications designed to prevent or halt the destruction of beta cells in individuals with newly diagnosed T1D (4). It allows the comparison of different interventions through the standardisation of data and sample collection within the clinical network. Importantly, this Master Protocol was discussed with the European Medicines Agency (EMA) during a qualification advice procedure to ensure that it meets the regulatory standards. Several phase II clinical trials linked with the Master Protocol are ongoing in children and adolescents, demonstrating the feasibility of implementing alternative innovative designs in paediatrics. The project also developed an adapted version of the INNODIA Master Protocol for studies in prevention. This Master Protocol, which received support from the EMA, aims to track T1D progression from the appearance of auto-antibodies, through dysglycaemia and to the full presentation with symptomatic disease (5). To pursue and maximise the impact of all these achievements, the international non-profit organisation INNODIA^5^ has been set up, providing a one-stop shop for all researchers to accelerate the development of treatment for people living with T1D.

The IMI project EU-PEARL^6^ developed a generic reusable framework and tools to facilitate the setup and implementation of patient-centric collaborative platform trials in Europe. As a prototype for paediatric rare diseases, the project developed a ready-to-operate platform trial in neurofibromatosis, with an observational phase to provide data on the natural course of the disease and a treatment phase to screen investigational agents for effectiveness in different manifestations of neurofibromatosis (6). The project successfully developed two master protocols—one for neurofibromatosis type 1 (NF1) and one for neurofibromatosis type 2 and schwannomatosis combined, enabling the Children’s Tumor Foundation to take forward and work further with the Global Coalition for Adaptive Research to implement the platform trial in NF1 and start soon to test new treatments.^7^

Leveraging EU-PEARL’s achievements, the newly started IHI project RealiseD^8^ will develop playbooks for further optimising clinical trial designs that could be used for any paediatric rare diseases.

### 2.3 Outcomes measures

Other challenges in conducting clinical trials in paediatrics include a poor understanding of the natural history of diseases, as well as a lack of appropriate biomarkers, endpoints and outcome measures. For instance, autism is a multifactorial and highly heterogeneous disorder, and this creates a challenge for running effective clinical trials for medicines and non-medical interventions. The IMI project AIMS-2-TRIALS^9^ focuses on better understanding how autism develops from before birth to adulthood. Candidate biomarkers that could potentially be used in a clinical trial context have been identified and are being progressed towards clinical validation and, ultimately, to regulatory qualification. A milestone was reached with the receipt of EMA letters of support for latency of the N170 event-related potential as a prognostic biomarker for adaptive social functioning (7) and for the VABS-II Adaptive Behaviour Composite score as a measure of adaptive social functioning (8). In addition to conducting high-quality clinical studies, the AIMS-2-TRIALS clinical network, consisting of 120 sites across 37 countries in Europe, is well-placed to test novel biomarkers and generate the necessary evidence to support their regulatory acceptance. For example, the project completed a phase II randomised trial that included the N170 biomarker as an exploratory measure (9).

## 3 Optimising paediatric cancer development

In the US, the Research to Accelerate Cures and Equity (RACE) for Children Act of 2017 introduced requirements for paediatric clinical trials for new oncology drugs with relevant molecular targets. In Europe, the proposed revision of the Paediatric Regulation widens potential development in children, based on the product mechanism of action (10). With this legislative evolution, almost all new cancer medicines for adults will have to be explored for potential paediatric indications. Having the right tools is therefore essential to preclinically prioritise which oncology products should be developed in paediatric populations.

The IMI project ITCC-P4^10^ was launched to deliver a sustainable translational paediatric preclinical testing platform in Europe. To support the selection, prioritisation, and planning of targeted molecule efficacy testing, the project developed a methodology for the determination of “target actionability” (11) and applied it to deliver several target actionability reviews (12, 13). The project also established over 450 patient-derived xenograft (PDX) models of the most common paediatric solid tumours, including models from relapses and rarer entities as well as liquid tumour models, and developed genetically engineered mouse models and organoids. Of all PDX models, over 350 have been fully characterised together with the matching patient’s tumours and blood. To guide the development of promising agents, the project released a white paper on the minimum preclinical proof-of-concept data packages to inform clinical development (14). To maximise the impact of ITCC-P4 in contributing to delivering precision medicine for childhood cancer patients and continuing to develop preclinical models, the ITCC-P4 gGmbH has been set up.^11^ Open to industry and any academic researchers, it provides access to laboratory models for systematic efficacy testing of compounds on fully characterised paediatric *in vivo* tumour models.

## 4 Improving diagnosis through screening

Early diagnosis is essential to maintain children’s health and minimise the risk of complications that may occur. Approximately 80% of rare diseases are of genetic origin, almost 70% of which present in childhood (15). Currently, it takes a long time for a rare disease to be diagnosed, thereby delaying management with appropriate treatment or care where treatments or active clinical research exist (16). The IMI project Screen4Care^12^ aims to shorten the path to rare disease diagnosis by designing a comprehensive genetic newborn screening framework that can be implemented into healthcare systems. The project is performing a systematic scoping of all significant initiatives on rare disease diagnosis and genetic newborn screening (gNBS) in Europe to establish a Screen4Care rare disease federated metadata platform. In addition, the project is carrying out “real life” gNBS in approximately 25,000 infants in Europe, using whole genome sequencing and customised, “ad-hoc” designed gene panels (17).

The new IHI project EDENT1FI^13^ is setting up a pan-European open platform to screen 200,000 children and adolescents to identify if they have early-stage T1D and are at risk of developing symptomatic T1D. Ultimately, by using specific markers, the project aims to find T1D early, refining how we detect it, managing it before symptoms appear, developing new treatments, and raising awareness.

## 5 Advancing childhood immunisation

Respiratory syncytial virus (RSV) is a leading cause of hospitalisation in infants. The IMI project RESCEU^14^ focussed on better understanding of the RSV disease burden, particularly in younger children (18). The project investigated the impact of RSV, including a prospective birth cohort study that showed that vaccinating pregnant women or healthy babies during their first winter season could reduce the healthcare burden caused by RSV (19). It also developed tools to better predict RSV outbreaks. All these results contribute to optimising public health responses to RSV and support planning for future RSV immunisation programmes. The IMI project PROMISE^15^ built further on RESCEU achievements. For instance, the project developed a simplified version of the RSV bronchiolitis severity score (ReSVinet score) that, once sufficiently validated, could be used not only as a clinical endpoint in clinical trials of therapeutics and vaccines but also to improve patient management (20). Both projects increased RSV knowledge and awareness, thereby helping the introduction of RSV vaccines in the EU for pregnant women, children, and older adults (21).

The IMI project PERISCOPE^16^ generated tools that contribute to accelerating the development of next-generation pertussis vaccines. These include new models and assays to predict and evaluate efficacy and safety and a better understanding of the immune response to pertussis vaccination and infection (22). The controlled human *Bordetella pertussis* infection model established in the project is already being used by researchers to evaluate the safety and efficacy of new pertussis vaccines (23).

## 6 Looking ahead

The development of medical products for the paediatric population presents unique challenges, mainly due to small patient populations, the characteristics of paediatric physiology and pathophysiology, a lack of appropriate preclinical models, and scientific and operational difficulties in designing clinical trials. The EU Paediatric Regulation has stimulated the development of medicines, and its revision aims to steer development in areas of unmet medical need.

Public–private partnerships such as IHI, leveraging academic and industrial knowledge and know-how, with strong parental and child involvement and regulatory engagement, play an important role in addressing complex public health and industrial challenges. IHI/IMI projects have boosted European paediatric research and successfully delivered a wide range of achievements. The outcomes are revolutionising paediatric development, and their full impact will be felt by children, their families, and wider society not only in Europe but also globally. Meaningful patient engagement in clinical research is essential, and in the examples cited above, the voices of children and their parents have been pivotal to ensuring that their needs were at the very centre of all research activities.

However, there are still research gaps and unmet needs to be addressed, for instance, in neonatal research. The development of paediatric medical devices and *in vitro* diagnostics also requires novel approaches to overcome technical and clinical challenges, such as the need to accommodate the devices to a wide range of sizes and physiological conditions that take into account children’s continuous anatomy and physiology changes as they grow (24).

The rapid evolution in medical science, coupled with emerging and converging technologies, creates opportunities for innovative approaches that could be beneficial to children. A concerted action is therefore needed to develop science-driven research and research infrastructures, as well as to ensure genuine engagement with children, young people, and families and support from key stakeholders including policymakers, regulators, and the broad child health community.

Building on IMI achievements, IHI is well-placed to further advance paediatric research. The cross-sectorial nature of IHI provides a unique platform for the pharmaceutical and medical technology industry sectors to work together with patients, academic researchers, healthcare providers, and regulators and address cross-cutting challenges to embrace the full spectrum of health technologies for the benefit of children.

In February 2024, IHI organised a Regulatory Science Summit to identify areas for future collaborative research that would contribute to regulatory science. One of the themes was paediatrics, where there is a clear common interest in stimulating innovation. The discussion identified several challenges, some of which could be a good fit for IHI as a cross-sectoral public–private partnership, such as a sustainable framework/infrastructure that could stimulate and speed up the paediatric development of medicines, diagnostics, and devices. Another subject that could be addressed by IHI is modelling how to scientifically operationalise the mechanism of action concept as proposed in the revision of the EU Paediatric Regulation. The meeting was an important first step to gather insights and ideas, and further discussions are needed to explore how to translate them into IHI project(s) that would deliver transformational results for all stakeholders (25).

More recently, the EMA released an updated list of Regulatory Science Research Needs (26), some of which relate to paediatrics such as research on pharmacological commonalities and research on modelling and simulation methods to support regulatory decisions. The IHI model would be well-placed to tackle these needs.

## 7 Conclusion

Significant progress has already been made in addressing paediatric needs; however, the development of paediatric medicines and devices lags behind. IHI public–private partnership model makes it uniquely suited to further support paediatric research and innovation, and some of its projects have already had a proven impact on drug development, regulatory science, and healthcare practise. At the halfway point of IHI, it is timely to focus our efforts on areas that not only would boost the competitiveness of Europe’s health research and innovation sector but, more importantly, will accelerate the development of innovative medicines, medical devices, diagnostics, and other interventions for the benefit of the whole paediatric population from neonates to adolescents.

## Footnotes

^1^ https://www.ihi.europa.eu/projects-results/project-factsheets/c4c

^2^ https://conect4children.org/2023/05/31/conect4childrenstichting

^3^ https://www.ihi.europa.eu/projects-results/project-factsheets/innodia

^4^ https://www.ihi.europa.eu/projects-results/project-factsheets/innodia-harvest

^5^ https://www.innodia.org/about-us

^6^ https://www.ihi.europa.eu/projects-results/project-factsheets/eu-pearl

^7^ Neurofibromatosis Platform Trial | Adaptive Clinical Trials for NF1

^8^ www.ihi.europa.eu/projects-results/project-factsheets/realised

^9^ https://www.ihi.europa.eu/projects-results/project-factsheets/aims-2-trials

^10^ https://www.ihi.europa.eu/projects-results/project-factsheets/itcc-p4

^11^ https://itccp4.com

^12^ https://www.ihi.europa.eu/projects-results/project-factsheets/screen4care

^13^ https://www.ihi.europa.eu/projects-results/project-factsheets/edent1fi

^14^ https://www.ihi.europa.eu/projects-results/project-factsheets/resceu

^15^ https://www.ihi.europa.eu/projects-results/project-factsheets/promise

^16^ https://www.ihi.europa.eu/projects-results/project-factsheets/periscope

## References

[1] Report from the Commission to the European Parliament and the Council State of Paediatric Medicines in the EU 10 years of the EU Paediatric Regulation COM (2017) 626. Available online at: https://health.ec.europa.eu/system/files/2017-11/2017_childrensmedicines_report_en_0.pdf.

[2] Owen J Sen A Aurich B Engel C Cavallaro G Degraeuwe E. Development of the CDISC pediatrics user guide: a CDISC and conect4children collaboration. Front Med. (2024) 11:1370916. doi: 10.3389/fmed.2024.1370916, PMID: 38966540 PMC11222900

[3] IMI FAIRplus project’s FAIR CookBooK. Available online at: https://faircookbook.elixir-europe.org/content/recipes/interoperability/c4c-clinical-trials.html.

[4] Dunger DB Bruggraber SFA Mander AP Marcovecchio ML Tree T Chmura PJ. INNODIA master protocol for the evaluation of investigational medicinal products in children, adolescents and adults with newly diagnosed type 1 diabetes. Trials. (2022) 23:414. doi: 10.1186/s13063-022-06259-z, PMID: 35585600 PMC9116021

[5] European Medicines Agency. Letter of support for Master Protocol for Type 1 diabetes prevention studies in INNODIA. (19 October 2021- EMADOC-1700519818-703684). Available online at: https://www.ema.europa.eu/en/documents/other/letter-support-master-protocol-type-1-diabetes-prevention-studies-innodia_en.pdf.

[6] IMI EU-Pearl. Project Deliverable 7.4: NF Master Protocol for IRPs. (2023). Available online at: https://cordis.europa.eu/project/id/853966/results.

[7] European Medicines Agency. Letter of support for N170 ERP as a prognostic biomarker for adaptive social functioning and its potential to stratify study populations in people with Autism spectrum disorders (ASD) without intellectual disability. (20 November 2020 -EMA/508140/2020). Available online at: https://www.ema.europa.eu/en/documents/other/letter-support-n170-erp-prognostic-biomarker-adaptive-social-functioning-and-its-potential-stratify-study-populations-people-autism-spectrum-disorders-asd-without-intellectual-disability_en.pdf.

[8] European Medicines Agency. Letter of support for the VABS-II Adaptive Behavior Composite (VABS-II-ABC) score as measure of adaptive social functioning in people with Autism Spectrum Disorders (ASD) without intellectual disability (20 November 2020 - EMA/548136/2020). Available online at: https://www.ema.europa.eu/en/documents/other/letter-support-vabs-ii-adaptive-behavior-composite-vabs-ii-abc-score-measure-adaptive-social-functioning-people-autism-spectrum-disorders-asd-without-intellectual-disability_en.pdf.

[9] Parellada M San José Cáceres A Palmer M Delorme R Jones EJH Parr JR. A phase II randomized, double-blind, placebo-controlled study of the efficacy, safety, and tolerability of arbaclofen administered for the treatment of social function in children and adolescents with autism Spectrum disorders: study protocol for AIMS-2-TRIALS-CT1. Front Psych. (2021) 12:701729. doi: 10.3389/fpsyt.2021.701729 PMC8421761 34504446

[10] Proposal for a Regulation of the European Parliament and of the Council laying down union procedures for the authorisation and supervision of medicinal products for human use and establishing rules governing the European medicines agency, amending Regulation (EC) no 1394/2007 and Regulation (EU) no 536/2014 and repealing Regulation (EC) no 726/2004, Regulation (EC) no 141/2000 and Regulation (EC) no 1901/2006 (COM/2023/193 final).

[11] Schubert NA Lowery CD Bergthold G Koster J Eleveld TF Rodríguez A. Systematic target actionability reviews of preclinical proof-of-concept papers to match targeted drugs to paediatric cancers. Eur J Cancer. (2020) 130:168–81. doi: 10.1016/j.ejca.2020.01.027, PMID: 32224415 PMC7203547

[12] Keller KM Krausert S Gopisetty A Luedtke D Koster J Schubert NA. Target Actionability review: a systematic evaluation of replication stress as a therapeutic target for paediatric solid malignancies. Eur J Cancer. (2022) 162:107–17. doi: 10.1016/j.ejca.2021.11.030 34963094

[13] Schubert NA Chen CY Rodríguez A Koster J Dowless M Pfister SM. Target actionability review to evaluate CDK4/6 as a therapeutic target in paediatric solid and brain tumours. Eur J Cancer. (2022) 170:196–208. doi: 10.1016/j.ejca.2022.04.028, PMID: 35671543

[14] Vassal G Houghton PJ Pfister SM Smith MA Caron HN Li XN. International consensus on minimum preclinical testing requirements for the development of innovative therapies for children and adolescents with Cancer. Mol Cancer Ther. (2021) 20:1462–8. doi: 10.1158/1535-7163.MCT-20-0394, PMID: 34108262

[15] The Lancet Global Health. The landscape for rare diseases in 2024. Lancet Glob Health. (2024) 12:e341. doi: 10.1016/S2214-109X(24)00056-1 38365397

[16] Faye F Crocione C Anido de Peña R Bellagambi S Escati Peñaloza L Hunter A. Time to diagnosis and determinants of diagnostic delays of people living with a rare disease: results of a rare barometer retrospective patient survey. Eur J Hum Genet. (2024) 32:1116–26. doi: 10.1038/s41431-024-01604-z, PMID: 38755315 PMC11369105

[17] Ferlini A Gross ES Garnier N on behalf of the Screen4Care consortium Berghout J Zygmunt A. Rare diseases' genetic newborn screening as the gateway to future genomic medicine: the Screen4Care EU-IMI project. Orphanet J Rare Dis. (2023) 18:310. doi: 10.1186/s13023-023-02916-x, PMID: 37794437 PMC10548672

[18] Li Y Wang X Blau DM Caballero MT Feikin DR Gill CJ. Michael et al. global, regional, and national disease burden estimates of acute lower respiratory infections due to respiratory syncytial virus in children younger than 5 years in 2019: a systematic analysis. Lancet. 399:2047–64. doi: 10.1016/S0140-6736(22)00478-0, PMID: 35598608 PMC7613574

[19] Wildenbeest JG Billard MN Zuurbier RP Korsten K Langedijk AC van de Ven PM. The burden of respiratory syncytial virus in healthy term-born infants in Europe: a prospective birth cohort study. Lancet Respir Med. (2023) 11:341–53. doi: 10.1016/S2213-2600(22)00414-3 36372082 PMC9764871

[20] Sheikh Z Potter E Li Y Drysdale SB Wildenbeest JG Robinson H. Louis Bont, Harish Nair, on behalf of the PROMISE investigators, external validation of the discriminative validity of the ReSVinet score and development of simplified ReSVinet scores in secondary care. J Infect Dis. (2024) 229:S18–24. doi: 10.1093/infdis/jiad388 37712125

[21] Bont L Demont C Nair H. RESCEU and PROMISE: the success of 8 years of European public-private partnership to prevent RSV. J Infect Dis. (2024) 229:S4–S7. doi: 10.1093/infdis/jiae012 38236160

[22] de Graaf H Ibrahim M Hill AR Gbesemete D Vaughan AT Gorringe A. Controlled human infection with *Bordetella pertussis* induces asymptomatic, immunizing colonization. Clin Infect Dis. (2020) 71:403–11. doi: 10.1093/cid/ciz840, PMID: 31562530 PMC7353841

[23] Keech C Miller VE Rizzardi B Hoyle C Pryor MJ Ferrand J. Immunogenicity and safety of BPZE1, an intranasal live attenuated pertussis vaccine, versus tetanus–diphtheria–acellular pertussis vaccine: a randomised, double-blind, phase 2b trial. Lancet. (2023) 401:843–55. doi: 10.1016/S0140-6736(22)02644-7, PMID: 36906345

[24] Espinoza J Shah P Nagendra G Bar-Cohen Y Richmond F. Pediatric medical device development and Regulation: current state, barriers, and opportunities. Pediatrics. (2022) 149:e2021053390. doi: 10.1542/peds.2021-053390, PMID: 35425971

[25] IHI Regulatory Science Summit 27-28 2024 Report. Available online at: https://www.ihi.europa.eu/sites/default/files/uploads/Documents/ProjectResources/RegulatoryScienceSummit_Feb2024_Report.pdf.

[26] Regulatory science research needs (EMA/510633/2024) 2024 update – DRAFT. Available online at: https://www.ema.europa.eu/en/documents/other/draft-regulatory-science-research-needs-2024-update_en.pdf.

